# Lotus petal flap for unusual indication: A recto-vaginal fistula with perineal defect after vaginal delivery^☆^

**DOI:** 10.1016/j.ijscr.2021.106337

**Published:** 2021-08-25

**Authors:** Ramzi Arfaoui, Mohamed Aymen Ferjaoui, Slim Khedhri, Kais Abdessamia, Mohamed Amine Hannechi, Khaled Neji

**Affiliations:** a The Maternity Department of Tunis Military Hospital, Tunis Medical School, El Manar University, Tunisia; b Department B of Gynecologic Surgery, Tunis Maternity Center, Tunis Medical School, El Manar University, Tunisia

**Keywords:** Obstetric tears, Perineal fistula, Fecal incontinence, Flap reconstruction

## Abstract

**Introduction and importance:**

Three to five percent of vaginal deliveries are complicated by third or fourth degree perineal laceration. Misdiagnosed perineal injuries may be associated with a poor sexual and psychological prognosis. Management of old perineal tears and laceration is challenging with a high failure rate. In such condition, interposition tissue technic or local flap can be a good surgical alternative. Lotus petal Flap, usually indicated in management of large perineal defect in gynecological oncology can be used.

**Case presentation:**

We report a case of 32-year-old women presenting complex and relapsed perineal fistula after vaginal delivery associated with perineal defect treated by lotus petal flap with a good outcome.

**Discussion:**

Perineal defects are commonly encountered after oncologic, traumatic or infectious perineal excisions and described as a challenging situation. In case of perineal defects after obstetrical tears, no validated surgical filler technics are recommended. Inspired from oncologic surgical technics to fill perineal defects, Lotus Flap can be used. Its advantages are to mobilize a satisfactory tissue volume to fill important perineal defect compared to the small bulbocavernous flap with a hidden scar comparing to gracilis muscle flap. This technic is associated with a good sexual and self-imaging outcome.

**Conclusion:**

Lotus petal flap may be required as a solution to manage perineal defect in case of perineal fistula. This technique provides aesthetic and good results for perineal reconstruction.

## Highlights

• Perineal defects secondary to obstetrical tears and injuries are challenging to manage.
• Many flaps and surgical technics were reported to cover perineal defects after oncologic perineal surgery.
• Lotus petal flap is one of the most used technics to cover perineal defects after oncologic perineal surgery.
• In literature, no recognized procedure was described to fill in perineal defects complicating obstetrical injuries.
• Lotus petal flap can be an efficient and safe procedure to cover perineal defects after obstetrical tears.

## 1 Introduction

Recto-vaginal fistula and external anal sphincter injuries occur in 3 to 5% of vaginal deliveries [1]. Misdiagnosed obstetrical injuries and perineal tears are related with fecal incontinence and pelvic organ prolapse [2]. It may impact the patient's fertility and sexual wellbeing. Many predisposing factors can lead to this obstetrical complication including instrumental delivery, fetal overweight, nulliparity and even maternal age [3]. The outcome of these perineal tears is poor due to local ischemia and inflammation. The recto-vaginal fistula relapses usually in 80% of cases [4]. Many surgical technics were described to manage such condition using tissue interposition and local flap [4], [5]. Lotus petal flap is performed usually to cover perineal defect after oncological procedure and to treat recto-vaginal fistula after colorectal surgery [6]. For perineal defects secondary to obstetric complications, this surgical technic is rarely reported.

In this case, we report a surgical procedure using lotus petal flap to cover recto-perineal defect associated with recto-vaginal and recto-perineal fistulas in a 32-year-old woman.

The work has been reported in line with the SCARE criteria [7].

## 2 Case report

A 32-year-old patient, without any medical history, gravida 1 para 1, delivered vaginally two years ago. She was referred to our department for fecal incontinence with smelly vaginal discharge. Clinical examination shows a low recto-vaginal fistula associated with hypotonic external anal sphincter. A surgery was decided. The patient underwent a recto-vaginal dissection, with running suture for fistula repairing. Puborectalis muscle plication and sphincteroplasty was performed.

**Fig. 1:**
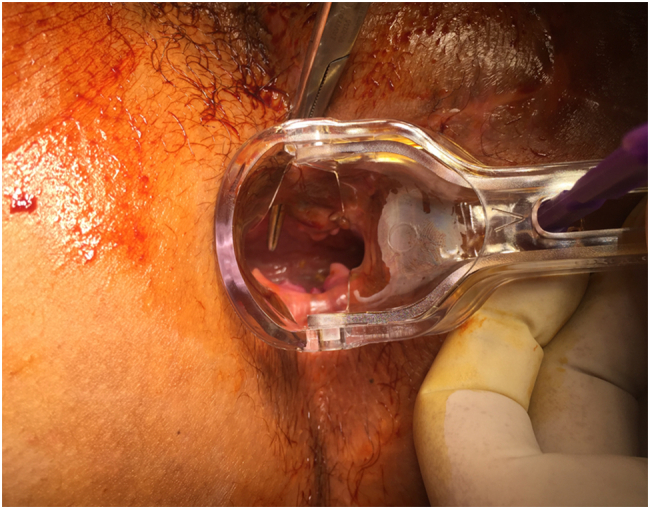
Anal retractor is used to show a low recto-vaginal fistula.

**Fig. 2:**
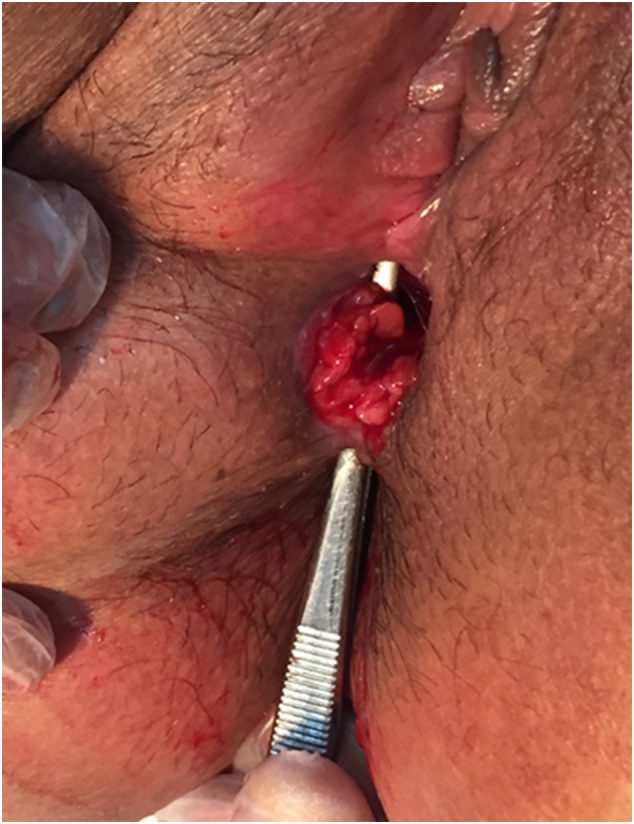
Recto-perineal fistula with a perineal defect.

Three months later, the patient presents a vaginal fecal discharge. Clinical examination shows a relapse of the abnormal recto-vaginal communication associated with a recto-perineal fistula and perineal defect (Fig. 1, Fig. 2). The recto-vaginal septum was thin. The surgical challenge was to treat the complex perineal fistula and to fill in the perineal defect.

**Fig. 3:**
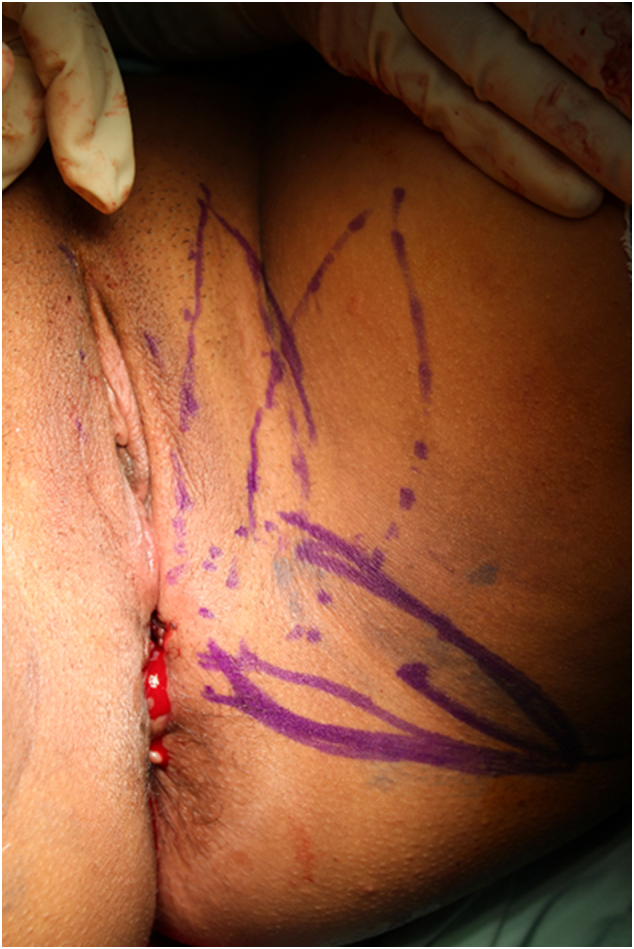
A marked lotus flap before surgery, patient was in lithotomy position.

**Fig. 4:**
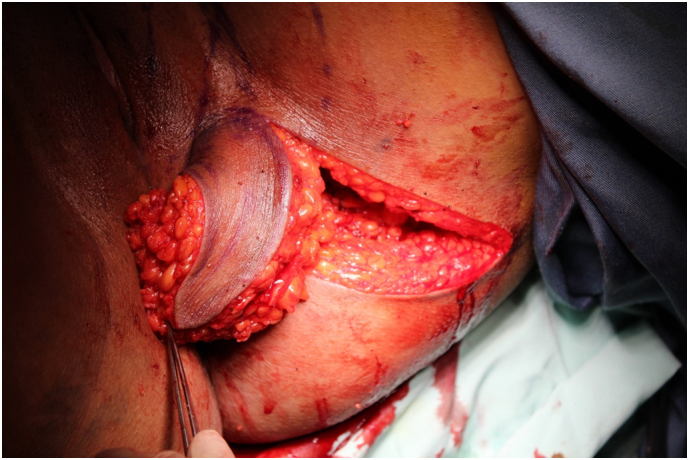
The flap rotation.

**Fig. 5:**
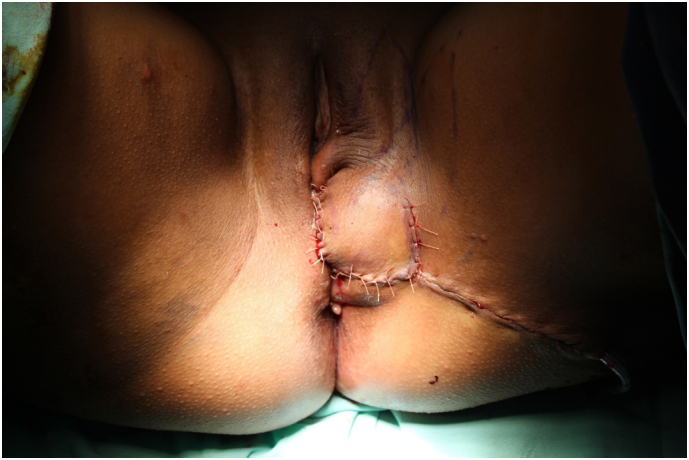
Post-operative aspect.

The use of lotus petal flap was decided. In lithotomy position and under general anesthesia, through a perineal rout, the recto-vaginal space was dissected. A large necrotic and infected tissue was removed. A lotus petal flap was performed and interposed to separate vaginal and rectal mucosa and to fill in the perineal defect (Fig. 3, Fig. 4, Fig. 5). The surgical procedure was uncomplicated and with uneventful outcome.

Six month later, she gets pregnant and an elective caesarian section was performed at 39 gestational weeks to avoid perineal complication.

## 3 Discussion

Perineal defects are commonly encountered after oncologic, traumatic or infectious perineal excisions and described as a challenging situation. Many surgical technics have been reported to repair these defects [8]. These procedures are generally associated with good prognosis, low complication rate and successful aesthetic results [9]. In case of perineal defect secondary to obstetric tears, the most reported flaps are gracilis muscle and bulbocavernous muscle flap but no validated surgical filler technics are recommended [3].

In our case, we manage the perineal defect complicating obstetrical perineal fistula with an unusual surgical filler procedure. We used lotus petal flap inspired from perineal oncologic technics to fill in obstetric perineal defect.

The fasciocutaneous lotus petal flap is a reconstructive technic used frequently to fill perineal oncologic defect [6]. It was described firstly in 1996 by Yii and Niranjan to reconstruct vulvar defect in gynecological oncology [10]. This fasciocutaneous flap is based on the important perineal vascular anastomoses. The key to perform the lotus petal flap is to localize the anatomical position of internal pudendal perforators arteries.

After vascular ultrasound determination, lotus petal flap can be drawn as a lotus flower around the located vessels and classified as inner, intermediate or lower petals according to their proximity to the introitus [11]. The flap is excised and modeled to cover the perineal defect. The donner site is closed and the lotus flap is sutured in layers. Surgically, the lotus flap dimensions are marked depending on location and size of the defect to fill. The width is marked to guarantee a free tension donor site closure. The length will be adjusted depending on defect dimension [10].

The advantages of lotus petal flap are to mobilize a satisfactory tissue volume to fill important perineal defect compared to the small bulbocavernous flap with a hidden scar comparing to gracilis muscle flap. This technic is associated with a good sexual and self-imaging outcome [12].

Fistula relapse is the most common complication of recto-vaginal fistula repair independently of used surgical procedure [4]. In this case, colostomy may be the solution to ovoid recurrence, which is not always required [10].

Although we performed lotus petal flap on one patient and we were satisfied with the outcomes. A prospective study needs to be done in order to have more accurate results and long term follow up.

The most local reported complications are infection and wound leakage due to high local bacteria count. These complications can be managed by local scar care.

## 4 Conclusion

Advanced obstetrical perineal injuries and lacerations are challenging to treat, especially with perineal defects. Filler reconstruction surgical technics have frustrating functional and aesthetic results. Lotus petal flap may be required as an efficient solution providing good outcome results.

## Funding

No funding resources to report.

## Ethical approval

This case report is exempt from ethical approval by our institution.

## Consent

Written informed consent was obtained from the patient for publication of this case report and accompanying images. A copy of the written consent is available for review by the Editor-in-Chief of this journal on request.

## Registration of research studies

This is not a “first in humans” report, so it is not in need of registration.

## Guarantor

Ferjaoui Mohamed Aymen

## Provenance and peer review

Not commissioned, externally peer-reviewed.

## Data sharing policy

The authors declare that data supporting the findings of this study are available within the article (and its supplementary files).

## CRediT authorship contribution statement

Arfaoui Ramzi: 1. Surgery and wrote the case report, 2. Revised and edited the case report, 3. Approved final version of case report.

Ferjaoui Mohamed Aymen: 1. Surgery and participated in patient care 2. Revised and edited the case report, 3. Approved final version of case report.

Slim Khedhri: 1. Participated in patient care 2. Revised and edited the case report, 3. Approved final version of case report.

Kais Abdessamia: 1. Revised and edited the case report.

Hannechi Mohamed Amine: Revised and edited the case report

Khaled Neji: approved final version of case report

## Declaration of competing interest

The authors report no declaration of competing interest.
